# Hearing loss: rising prevalence and impact

**DOI:** 10.2471/BLT.19.224683

**Published:** 2019-10-01

**Authors:** Adrian C Davis, Howard J Hoffman

**Affiliations:** a The Ear Institute, University College London, 332 Grays Inn Rd, Kings Cross, WC1X 8EE, London, England.; b Division of Scientific Programs, National Institute on Deafness and Other Communication Disorders, Bethesda, United States of America.

The global prevalence of sensorineural hearing impairment was first reported by the World Health Organization (WHO) in 1985.^1^ At that time, 42 million people (approximately 1% of the world’s population) were estimated to have moderate to profound (or disabling) hearing impairment. By 2011, the estimate rose to 360 million, of whom 32 million were children younger than 15 years.^2^ The most recent WHO estimate suggests that approximately 466 million people (or 6.1% of the world’s population) were living with disabling hearing loss in 2018.^3^ This estimate is projected to rise to 630 million by 2030 and to over 900 million by 2050. Approximately 90% of people with moderate to profound hearing impairment reside in low- and middle-income countries. The Global Burden of Disease study, which incorporated mild and unilateral hearing loss, estimated that the population with hearing loss rose from 1.2 billion (17.2%) in 2008 to 1.4 billion (18.7%) in 2017.^4^ This trend has become a serious public health issue that deserves an appropriate and well-coordinated global action.

At the individual level, the burden of hearing loss over the life course is substantial and can be exacerbated by negative societal attitudes and prejudice towards affected people. In general, hearing loss has adverse consequences on interpersonal communication, psychosocial well-being, quality of life and economic independence.^5^^–^^7^ The condition impedes speech and language development from early childhood and can set affected children on a trajectory of sub-optimal educational and vocational attainment. Adults with hearing loss often experience social isolation and stigmatization, abuse, psychiatric disturbance, depression, difficulties in relationships with partners and children, restricted career choices, occupational stress and relatively low earnings.^8^ The economic impact of unaddressed hearing loss on health, education and productivity for society is staggering, estimated to cost over 750 billion United States dollars globally.^9^ This figure is expected to rise as the number of people with hearing loss rapidly increases across the world.

The main drivers for the rising trend in hearing loss include demographic changes attributable to global population growth and improved life expectancy.^3^^,^^5^^,^^10^ The substantial global investments on child survival programmes since 2000 have also resulted in a growing population of beneficiaries with special health-care needs in low- and middle-income countries.^6^ Sensorineural hearing loss, which results from damage to the hair cells in the inner ear, is more frequently considered as the major pathway to permanent hearing impairment. Many countries lack programmes to reduce exposure to risk factors such as occupational and recreational noise. The use of ototoxic drugs needs to be minimized.^5^ Significant causes of increased prevalence include, common ear conditions such as chronic otitis media with effusion and vaccine-preventable infections such as measles, mumps, rubella and bacterial meningitis.^10^ A large proportion of the incidence of hearing loss could be prevented through appropriate interventions including community-oriented health education.

Since 2007, WHO has promoted increased public awareness of hearing loss through World Hearing Day, held every March. Actions required to curtail the growing number of people with hearing loss and to improve quality of life for those with hearing loss have also been extensively reported.^3^^,^^5^^,^^10^ However, progress is still limited, especially in low- and middle-income countries, due to insufficient local capacity to scale up proven interventions at all levels of health-care delivery. This shortcoming may be partly attributed to the lack of a robust global initiative and funding support for hearing health care. For example, the screening of newborns for possible hearing loss is routinely offered in most high-income countries but does not appear to be considered as necessary in low- and middle-income countries. Yet, the needed investment on low-cost hearing technologies for early detection and rehabilitation of people with hearing impairment is likely to be outweighed by the overall long-term benefits of their economic participation in society.^7^^,^^9^

The sustainable development goals explicitly recognize the need for a better quality of life and opportunities for optimal well-being throughout the life course of all people with disability.^11^ With the World Health Assembly’s resolution on the prevention of deafness and hearing loss^2^ and the Convention on the Rights of Persons with Disability,^12^ the needed global policy framework for curtailing the growing burden of hearing loss is now in place. To monitor progress meaningfully, global initiatives for hearing health care must be accompanied by specific, measurable and time-bound targets to reduce the number of people with hearing loss.
